# Living Donor Liver Transplantation as a Backup Procedure: Treatment Strategy for Hepatocellular Adenomas Requiring Complex Resections

**DOI:** 10.1155/2022/1015061

**Published:** 2022-02-17

**Authors:** Eduardo A. Fonseca, Flavia Feier, Rodrigo Vincenzi, Helry L. L. Candido, Rodrigo L. Azambuja, Fabio Payao, Marcel R. Benavides, Karina M. O. Roda, Katia M. R. Leite, Cristiane M. F. Ribeiro, Maria D. Begnami, Charles E. Zurstrassen, Francisco C. Carnevale, Paulo Chapchap, João Seda-Neto

**Affiliations:** ^1^Liver Transplant Unit, Hospital Sirio-Libanes, São Paulo, Brazil; ^2^Liver Transplant Unit, A.C. Camargo Cancer Center, São Paulo, Brazil; ^3^Liver Transplant Unit, Santa Casa de Porto Alegre, RS, Brazil; ^4^Department of Radiology and Imaging, Hospital Sirio-Libanes, Brazil; ^5^Department of Pathology, Hospital Sirio-Libanes, Brazil; ^6^Department of Pathology, A.C. Camargo Cancer Center, Brazil; ^7^Vascular and Interventional Radiology Department, A.C. Camargo Cancer Center, Brazil; ^8^Vascular and Interventional Radiology Section, Hospital Sirio-Libanes, Brazil

## Abstract

**Methods:**

We present a series of three patients with large hepatocellular adenoma lesions showing a central location, for which the living donor liver transplantation strategy was used as a backup procedure.

**Results:**

Hepatocellular adenoma was confirmed by biopsy in all patients. Surgical resection was indicated because of the patients' symptoms and lesion size and growth. All patients had a lesion that was central or in close contact with major vessels. The final decision to proceed with the resection was made intraoperatively. A live donor was prepared for all three patients. Two patients underwent portal vein embolization associated with extended hepatectomy, and a total hepatectomy plus liver transplantation with a living donor was performed in one patient. All patients had good postoperative outcomes.

**Conclusions:**

In the treatment of hepatocellular adenomas for which complex resections are necessary and resectability can only be confirmed intraoperatively, surgical safety can be improved through the use of a living donor backup. Center expertise with living donor liver transplantation is paramount for the success of this approach.

## 1. Introduction

A hepatocellular adenoma (HCA) is a benign tumor that typically develops in a healthy liver. This condition usually occurs in women between 15 and 45 years of age and is associated with the use of oral contraceptives [1, 2]. The most dangerous complications associated with HCA are hemorrhage and malignant transformation, both of which require surgical treatment [3, 4].

Liver transplantation (LT) is an alternative for patients with HCA or liver adenomatosis deemed unresectable, or when other treatment strategies have failed. However, large or central tumors usually require complex hepatectomies, with the potential risk of damaging the future liver remnant (FLR). Having a back-up living donor for transplantation can inform the most appropriate strategy for extreme resections.

We report a series of three patients with large HCA who were taken to the operating room with a living donor prepared for a backup procedure in case resectability was not possible.

### 1.1. Patient One

A 12-year-old girl, weighing 22 kg, presented with a 2-year history of pruritus and a right upper quadrant mass. The patient's history did not include the use of hormones, and she did not present with jaundice, fecal acholia, or coluria. All laboratory liver tests and alpha-fetoprotein (AFP) levels were normal. Underling liver disease was ruled out.

**Figure 1:**
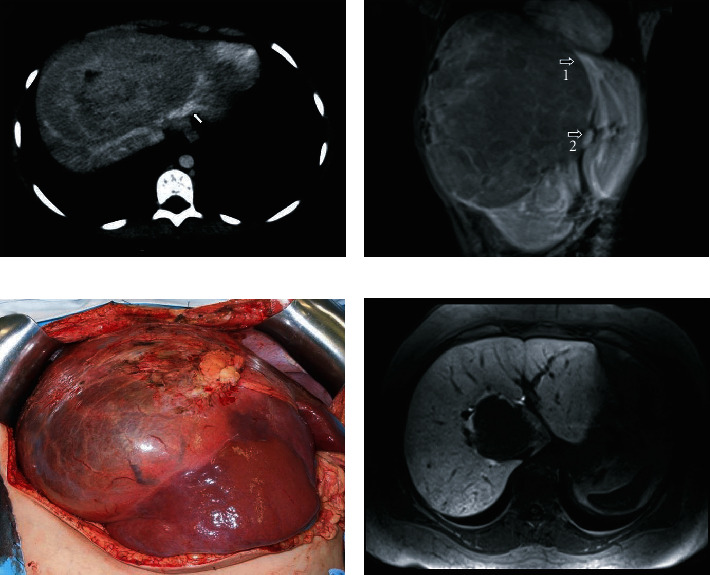
(a) MRI images showing a large heterogeneous mass compromising the RL and segment IV and in close contact with the FLR vessels (Arrow). (b) MRI image showing a large heterogeneous lesion occupying the whole RL and preserving segments II, III, and inferior IV. The left hepatic vein (arrow 1) and the left branch of the portal vein (arrow 2) are in close contact with the lesion. (c) Intraoperative aspect: tumor mass occupying almost the entire liver, preserving only segments II, III, and inferior IV. (d) MRI with hepatospecific contrast agent showing a lesion without contrast enhancement on the hepatobiliary phase in the caudate lobe with involvement of the middle and right hepatic veins and in close contact with the left hepatic vein.

Magnetic resonance imaging (MRI) showed a large heterogeneous hypointense mass measuring 23 cm × 15.3 cm × 12.4 cm that compromised the right lobe (RL) of the liver, segments IV and I (Table 1). Additionally, the mass was in close contact with the FLR vessels (Figure 1(a)). FLR volume was of 105 cm^3^, representing 14% of the total liver volume (TLV), and 0.47% of the patient weight ratio.

A liver biopsy of the mass was performed and showed features compatible with HCA, and the immunohistochemical (IHQ) result showed the HNF1-mutated subtype.

A right portal vein embolization (PVE) was performed to increase the FLR, thus allowing the extended hepatectomy.

On the third day after PVE, the patient presented with abdominal pain, hypotension, and a drop in serum hemoglobin concentration. Computerized tomography (CT) suggested intralesional active arterial bleeding, and the patient underwent a transarterial embolization. A new CT scan was performed seven weeks later and showed an FLR of 504 cm^3^, which corresponded to 40% of the TLV and 2.3% of the patient weight ratio.

Due to the close contact of the mass with major vessels, the assessment of potential living related donors for a backup procedure was performed. A 35-year-old female, a related family member who was ABO compatible, was selected as a living donor and prepared according to a previously published protocol [5].

An extended right hepatectomy with caudate resection was performed in March/2016, and the entire tumor was removed with no need to proceed with the LT. Histopathological analysis confirmed the diagnosis and had no signs of malignant transformation. The patient has been followed for 5 years since surgery and is clinically well, with normal liver function tests. A follow-up CT scan showed no residual tumor.

### 1.2. Patient Two

A 12-year-old girl, weighting 28 kg, presented with a six-month history of pain and abdominal growth associated with vomiting and progressive shortness of breath, featuring abdominal compartment syndrome.

Liver function tests and serum AFP levels were normal. Underling liver disease was ruled out.

MRI showed a large heterogeneous lesion measuring 20 cm × 16 cm × 16 cm occupying the entire RL and preserving segments II, III, and inferior IV (Table 1). The tumor involved the right and medium hepatic veins, as well as segment IV and the right branches of the portal vein (PV). The left hepatic vein and the left branch of the PV had close contact with the lesion (Figure 1(b)). Estimated FLR volume was of 180 cm^3^, which represented 28% of the TLV and 0.64% of the patient weight ratio.

A liver biopsy was performed and showed features compatible with HCA, and the IHQ analysis identified an inflammatory subtype.

Because of the close contact of the mass with major vessels, a back-up live donor was evaluated. The donor was a healthy 31-year-old female, ABO compatible. The estimated volume of the left liver was of 450 cm^3^, which corresponded to 33% of the TLV and a graft to recipient weight ratio (GRWR) of 1.5%.

The tumor mass occupied almost the entire liver, preserving only segments II, III, and IVb (Figure 1(c)). The intraoperative ultrasound demonstrated left PV involvement, compromising the FLR inflow. Based on these intraoperative results, the decision was made to abort the planned liver resection, and a total hepatectomy followed by living donor liver transplant (LDLT) was performed in January/2012.

A left liver graft was implanted with a cold and warm ischemia time of 55 and 25 minutes, respectively. The operation lasted 10 hours. Both the donor and recipient postoperative courses were uneventful, and the patients were discharged after 6 and 11 days, respectively. Histopathological analysis confirmed the diagnosis and did not demonstrate signs of malignant transformation. Currently, the recipient is in the tenth year of follow-up, clinically stable, and with normal liver tests.

### 1.3. Patient Three

A 40-year-old female, weighting 95 kg, presented with an eight-month history of abdominal pain. Oral contraceptives had been used for 20 years.

On physical examination, the patient was overweight, with a body mass index of 32.5 kg/m^2^. Liver function tests and serum AFP levels were normal. Underling liver disease was ruled out.

An abdominal MRI showed the absence of underlying chronic liver disease and a solitary lesion with HCA characteristics measuring 4.6 cm in the caudate lobe.

The patient was instructed to discontinue oral contraceptives, lose weight, and repeat the imaging exams. After eight months, an abdominal MRI showed a solid hypervascularized lesion measuring 9.3 cm (Table 1), with involvement of the middle and right hepatic veins and close contact with the left hepatic vein (Figure 1(d)). The hepatic volumetry showed an FLR (II and III) of 291 cm^3^, corresponding to 12.5% of the TLV and 0.31% of the patient weight ratio. The patient underwent a percutaneous biopsy, which revealed an HCA-inflammatory subtype. PVE of the right and segment IV portal branches was performed through a percutaneous ipsilateral approach. No complications were observed after PVE. CT was performed 6 weeks later, with an FLR volume of 761 cm^3^, corresponding to 32.7% of the TLV and 0.82% of the patient weight ratio.

Due to the close proximity of the tumor with the FLR left hepatic vein, an evaluation and work-up of a potential live donor occurred as part of a backup plan.

A 19-year-old male who was ABO compatible was selected as a live donor (the patient's son). General tests used for donor preparation were normal.

The patient underwent surgical resection in September/2015, and the remnant left liver was macroscopically normal. Intraoperative ultrasound showed no involvement of the left hepatic vein by the tumor. Extended hepatectomy with caudate resection was performed. Histopathological analysis confirmed the diagnosis and showed no signs of malignant transformation. Currently, 6 years after surgery, she is in good general condition with normal liver function tests. An abdominal MRI was performed fifteen months after the operation and showed a hypertrophied left liver without residual tumor.

## 2. Discussion

Recent advances in imaging as well as the acquisition of new tools in molecular biology to complement the diagnosis of HCA subtypes [6, 7] have contributed to a greater understanding of the evolution of these tumors and consequently to the development of good clinical practices for their management.

The surgical approach is recommended for asymptomatic patients with the following HCA conditions: larger than 5 cm, harboring hepatocellular carcinoma or dysplastic foci, *β*-catenin activated, increasing size or imaging features of malignant transformation, rising AFP, males, and glycogen storage disease [8–10].

Symptoms of pain and a large liver mass led to the decision to proceed with surgery in the patients described herein. The fact that the lesion was benign made the decision to proceed with surgery more difficult, because the central location of the tumors creates a technical challenge.

The management of patients with HCA is a controversial topic. A few publications and small patient series report complications in the evolution of these patients. Hemorrhage is the most prevalent complication in published studies and shows a direct correlation with the size and the superficial location of these lesions [3, 4]. There is a tendency in the literature to recommend LT for patients with adenomatosis for whom the surgical resection is complex [11, 12]. The largest report on LT for adenomatosis is from the European group: of 49 patients with histologically confirmed adenomatosis (33% with GSD), 17 (34.6%) patients presented with HCC in liver explants, and a total of 8 (16.3%) patients died after LT during follow-up [13]. Chiche et al. reported a follow-up in 8 patients with adenomatosis; in 2 of them, liver transplantation was necessary due to symptoms of pain and hepatomegaly [14]. In patients with large tumors (isolated or multiple) or with tumors that are in close proximity to the vascular structures, resection is associated with perioperative morbidity and eventual mortality. These outcomes are related not only to the proximity of the tumors to the hepatic inflow and outflow vessels but are also associated with the small FLR and the potential for postoperative liver failure.

It was reported in the literature that patients who received LT for unresectable HCA typically showed a small FLR at the time of resection [12]; however, LT must be reserved for those patients with irrefutable unresectable tumors, because of the side effects associated with a lifetime of immunosuppressive therapy. Therefore, surgical resection remains the mainstay of curative treatment.

Currently, the clinical use of techniques that allow for compensatory hypertrophy of the FLR, such as PVE, has become pivotal in preparing for major liver resections [15–17], preventing posthepatectomy liver failure in patients with a small FLR [18, 19]. In those patients (patients 1 and 3) who underwent PVE, an increase in the FLR volume was observed which enabled total resection of the tumor to be performed safely.

However, PVE is an invasive procedure that is not free of risks. The most common complication of percutaneous transhepatic procedures is hemorrhage, and its occurrence has been reported in 2-4% of patients. When there is massive bleeding, transarterial embolization is the most effective treatment, as occurred in patient 1 [20].

All patients in this series were submitted to surgical treatment, and a living donor served as a backup strategy because of uncertainties regarding tumor resectability and the viability of the FLR. Although the patients who underwent PVE presented with a final increase in the size of the FLR sufficient for their metabolic demands after surgery, a living donor was used as a backup strategy because of the close proximity of the lesion to the vessels of the FLR. In both patients, complete lesion resection was achieved without the need to use the living donor.

LDLT is more appropriate for this option because the procedure can be scheduled. In potentially resectable large tumors, this strategy adds a particular benefit because of potential technical difficulties that may arise during tumor resection, such as a delay or prolongation in cold ischemia time when grafts are used from deceased donors, which often precludes the success of the LT. In this series, only one of the patients actually underwent LT (patient 2), who presented evidence of unresectability because of vascular involvement of the FLR. The living donor backup procedure becomes viable only for medical groups with proven experience in complex liver resection and LDLT. Our group has extensive experience with LDLT, with no procedure-related mortality and a low incidence of severe complications [5].

Thus, the use of the living donor backup procedure should be part of the treatment strategy in complex resections for benign lesions when the resectability is unclear, ensuring the safety of the liver resection.

## 3. Conclusion

HCA is a benign disease, and there are challenging aspects to the surgical treatment strategy for patients with large and central HCAs that require complex liver resections. Extended hepatectomy linked to the donor backup procedure provides security in complex liver resections and should become part of the surgical treatment approach for these patients. Additionally, this approach should be performed in centers with personnel who have expertise in hepatobiliary surgery and liver transplantation using a living donor.

## Figures and Tables

**Table 1:** Patients' characteristics.

	Patient 1	Patient 2	Patient 3
Gender, age (Y)	Female, 12	Female, 12	Female, 40
Tumor diameter (cm)	23	20	9.3
Liver segments involved	I, IV, to VIII	IVa, V, to VIII	I, V, to VIII
FLR (%)	14	28	12.5
PVE	Yes	No	Yes
Treatment	Extended right hepatectomy	LDLT–back-up	Extended right hepatectomy
Follow-up	5 y	10 y	6 y

FLR: future liver remnant; PVE: portal vein embolization; LDLT: living donor liver transplantation.

## Data Availability

Data is available upon request to the corresponding author.
